# Periodontal index and salivary Ph in first trimester´s pregnant women: A cross-sectional study

**DOI:** 10.4317/jced.60522

**Published:** 2024-04-01

**Authors:** Rocío-Paola Lozano, Angel-Steven Asmat-Abanto

**Affiliations:** 1 Dental Surgeon from Stomatology’s Study Program - Antenor Orrego Private University, Trujillo, Peru. Diploma in Basic Orthodontics. Diploma in Restorative and Aesthetic Dentistry; 2 Professor of the Human Medicine Program of the Antenor Orrego Private University. Trujillo-Peru. Professor at the Stomatology’s Study Program at the Antenor Orrego Private University. Trujillo-Peru. Doctor in Stomatology. Specialist in Periodontic

## Abstract

**Background:**

Recent reviews have suggested a possible association between periodontal disease and increasing risks during pregnancy, such as newborn low weight, premature birth, gestational diabetes mellitus and preeclampsia. Besides, being saliva a valuable source of information on general and buccal health, it is necessary to know its parameters associated with periodontal diseases; in order to help odontologists to better understand this disease, and provide a sound clinical treatment. Therefore, this research sought to assess the correlation between periodontal index, and salivary pH on first trimester pregnant women.

**Material and Methods:**

71 pregnant women were evaluated by observational, transversal and correlational study, using the community periodontal index and salivary pH-meter. The Spearman coefficient was employed, considering a p value of 0.05.

**Results:**

A correlation was found between periodontal index and salivary pH in first trimester pregnant women (*p*=0.039). According to age, correlations were found to be very weak in age group 18 to 24 years (*p*=0.605), and age group 25 to 35 years (*p*=0.071). Similarly, no strong correlation was found when pairing based on number of pregnancies (primiparous *p*=0.239 and multi pregnancy *p*=0.114). Also, 36.6% of pregnant women showed periodontal pockets smaller than 5 mm.

**Conclusions:**

With lower salivary pH, the periodontal index in pregnant women gets lower; however, no correlation could be found between age group and number of pregnancies.

** Key words:**Periodontal index, periodontal pocket, periodontal diseases, saliva, pregnancy, pregnant women.

## Introduction

Periodontal disease (PD) is a chronic infection affecting tissue surrounding teeth. When inflammation is restricted to gums is called gingivitis, usually caused by inadequate oral hygiene and; if not properly treated, could develop into periodontitis (1,2). PD is initiated by excessive growth of certain bacterial species, mostly Gram-negative, located in subgingival sites (3).

Human saliva is a complex fluid produced by salivary glands. It is composed mostly of 99% water, and 1% inorganic and organic content. Serve several functions including initial digestion process, bacterial elimination, lubrication of mucosal tissue, speech, and cleansing and protection of mouth cavity, through pH maintenance and buffering (4,5). As such, saliva plays an important role in keeping mouth cavity healthy. Changes in saliva production or composition will negatively affect local and general health of the individual (6).

During pregnancy, mother and developing baby will experience physiological changes that affect different organs, alteration of immune system increasing susceptibility of the mother to various infections, including infections to the mouth cavity. These can occur because of a reduction in pH or saliva production, favoring the growth and metabolism of acidogenic bacteria. Several studies indicate an increase in saliva production among pregnant women, occurring mainly during the first trimester. Usually, pregnant women exhibit nausea, vomiting and/or acidity in the stomach, which could trigger further changes in the mouth cavity, increasing the risk of caries and susceptibility to periodontal inflammation. In regular pregnancies, associated susceptibility to gingivitis occurs between week 12 and 28 of pregnancy (6-12).

Recent studies suggest placenta could harbor a unique microbiome that may have its origin in the mother oral microbiome. Although the large physiological and hormonal adjustments observed in pregnant women lead to biochemical and microbiological modifications in the oral cavity; the maternal oral microbiome may represent a main contributor to intrauterine microbiome, and yeast may affect fetus development and outcome of pregnancy. Growth of yest in the oral cavity during pregnancy may be associated with observed reduced pH in oral cavity in pregnant women. Following this suggestion, studies show that *Candida*, the most prevalent yeast in oral cavity, is better adapted to acidic conditions (13).

Several recent reviews have suggested a possible association between periodontal disease and increasing risks during pregnancy, such as newborn low weight, premature birth, gestational diabetes mellitus and preeclampsia (14-17). Besides, saliva is a valuable source of information on general and buccal health. It is necessary to know the parameters associated with periodontal diseases to help odontologists better understand this disease and provide a sound clinical treatment. Therefore, this research sought to assess the correlation between periodontal index, and salivary pH on first-trimester pregnant women; and contribute to the understanding of risk factors and protection measures for periodontal disease in first trimester pregnant women as vulnerable group.

## Material and Methods

The following research complies with an observational, transversal and correlational design, and was performed at the Obstetric Services area of Bellavista Health Care Center, La Libertad Region, Peru; from July to August, 2019.

Sample size was estimated using formula for estimation of frequencies with known population size. Sample included 71 patients. For this, a monthly attention to 120 pregnant women was considered, with 5% precision, 97.5% confidence level, and 5% type I error. Also, a pilot study indicated for code 0 a value of 12.5%. This sample was selected using non-probability accidental method.

Patients from first trimester pregnancy were included in the study, with good general health condition, age between 18 and 35 years, and who had not eaten at least for two hours prior to sample take. Patients who did not wish to participate were excluded from the study, as well as those who were taking antibiotics, immunosuppressants, antihypertensive drugs, anticonvulsants, and antidepressants.

Data collection was separated in two parts: first included filiation data and general information, including age and number of previous pregnancies, the second included records of salivary pH and Community Periodontal Index (CPI).

For this index the periodontal condition is evaluated with a 0,5 mm round tip periodontal probe, considering 10 teeth in the oral cavity; and subsequently assessed the appearance of gum bleeding, supragingival and subgingival calculus, periodontal bags, as well as loss of clinical insertion. Although this system analyzes a limited number of teeth, it has been shown to be representative of full mouth records. 18

Method reliability was obtained through inter and intra-evaluator calibration in a pilot study, where 32 patients were examined, participating as gold standard a university professor with 8 years of experience as Periodontist. Cohen’s kappa (κ) statistic was used to assess the reliability of “periodontal index” measurement. A value of k=0.780 was obtained from inter-evaluator calibration, and k=0.866 from intra-evaluator calibration.

Reliability of pH-meter (900 amps Digital RoHs pH meter) for saliva samples, was established through calibration by a certified technician from Bellavista Health Care Center, which belongs to Peru Ministry of Health.

This study was authorized by the Faculty of Human Medicine, and by the Bioethics Committee of Antenor Orrego Private University, through Resolution N° N° 240-2019-UPAO. The study was also authorized by the Director of Bellavista Health Care Center. These authorizations considered the Ethical Principles for Medical Research Involving Human Subjects stated in the Declaration of Helsinki from the World Medical Association, its more recent version as considered in Peru General Health Law N° 26842.

Each selected patient received information regarding the purpose of the research study, asking for her participation. Upon acceptance, the patient received the informed consent format for reading and signature. Later, saliva samples were obtained and periodontal clinic evaluation was performed.

For sampling, the patient was asked to fill the mouth with saliva and spit into a sterile container, and measurement was made using a pH meter for 5 seconds; values were recorded and noted in data collection forms. All saliva samples were collected at the same time during the days of the study. The assessment of the patient periodontal index was made through clinical examination in the Odontology Service at Bellavista Health Care Center. Artificial light was used, and the periodontal screening using a Hu-Friedy 11.5B WHO style probe. Values were recorded in data collection forms.

Data obtained was automatically processed using Microsoft Excel sheets and the program SPSS Statistics 22.0 (IBM, Armonk, NY, USA). Results were presented using Tables or graphs, according to stated research goals. To assess the correlation between periodontal index and saliva pH, the Spearman Correlation Index (Rho) was used, with a 5% significance level.

## Results

At the Obstetrics service area of Bellavista Health Care Center, La Libertad Region, Peru, roughly 120 pregnant women are treated every month. The Health Center offers a free program of buccal health assistance for pregnant women, including dental cleaning and four dental restorations during pregnancy.

In the present study, 71 pregnant women were evaluated, from 18 to 35 years of age (average age = 25.9 years, SD=0.06), who complied with study requirements as stated in the previous section. The following results were obtained:

In pregnant women include on the study, saliva pH varied from 4.72 to 8. Also, regarding Community Periodontal Index (CPI), code 3 was prevalent; that is, 36.6% of pregnant women presented periodontal pockets smaller than 5.5 mm. Also, lower frequencies of code 1 (hemorrhagic gingivitis), and code 4 (periodontal pockets smaller larger than 5.5 mm) were recorded, both with 18.3% (Table 1).

**Table 1:**
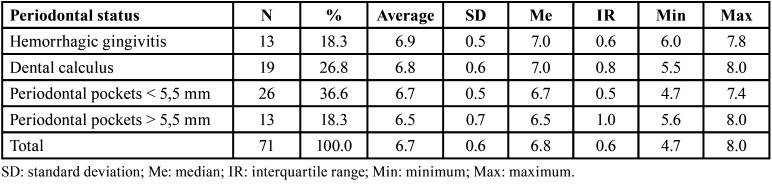
Periodontal index and salivary pH in first trimester´s pregnant women of Bellavista Health Care Center, La Libertad Region, Peru.

A correlation was found between Community Periodontal Index and saliva pH in first trimester pregnant women (*p*=0.039) (Table 2): The lower the pH, the worse the Community Periodontal Index. However, when age groups were analyzed, no correlation was found for age group 18 to 24 years (*p*=0.605) and for age group 25 to 35 years (*p*=0.071) (Table 3). Also, no correlation was found in relation to number of pregnancies, with *p*=0.239 for primiparous patients, and *p*=0.114 for multigestation patients (Table 4).

**Table 2:**
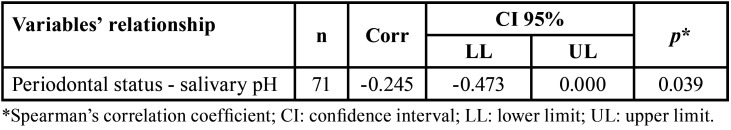
Relationship between periodontal index and salivary pH in first trimester´s pregnant women.

**Table 3:**
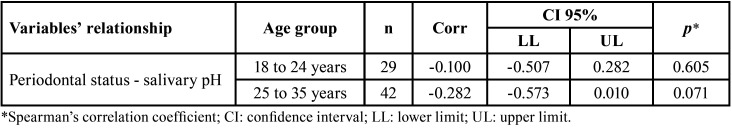
Relationship between periodontal index and salivary pH in first trimester´s pregnant women, according to age group.

**Table 4:**
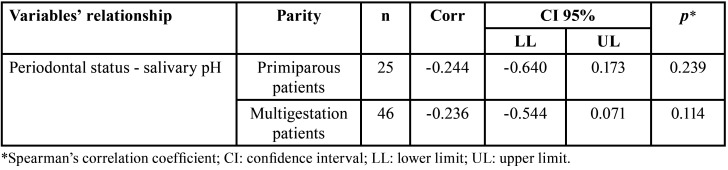
Relationship between periodontal index and salivary pH in first trimester´s pregnant women, according to parity.

## Discussion

Several studies relate periodontal disease with adverse effects during pregnancy (16,17) as well as the influence of saliva pH in mouth health maintenance (6); hence the importance of the study of possible relationships between these factors.

During the present study it was observed that patients with lower saliva pH also exhibited higher deterioration of periodontal condition. This probably is due to periodontal disease being influenced by the amount of dental biofilm, and this biofilm easily develops at lower pH values (19).

Development of dental biofilm is the primary cause and determinant factor in pregnancy gingivitis, therefore oral hygiene is considered an important factor in saliva pH variation, and periodontal condition (6).

Regarding the periodontal index, it was found that PCI code 3 was the most frequent in pregnant women evaluated (periodontal pockets smaller than 5.5 mm). These results are similar to those obtained by Chen *et al*. (20), Chuan-Che *et al*. (21) and Meena *et al*. (22), who used the same method to assess the probe depth and determine periodontal index. These periodontal pockets could be real or “periodontal pseudopockets”, owing to edema or gingival enlargement, resulting in the accumulation of bacterial plaque to high levels and, with time, could destroy the other components of the periodont. These complications must be treated opportunely to avoid progress or development of more severe forms of the disease. In pregnant women, these conditions could become a risk factor leading to premature birth, low weight of newborn, and preeclampsia (16,17).

At assessing saliva pH values, it was found 4.7 as lower value, and 8 as highest value. These findings do not agree with those of Kamate *et al*. (23), who found 6.8 as lower and 7.7 as higher pH values. This discrepancy is probably due to the lower number of people included in the aforementioned study, as well as differences in diet among each country.

According to age group and number of pregnancies, no correlation was found between periodontal index and saliva pH. This is probably due to the fact that, grouped according to these variables, population size was diminished, challenging statistical results. Also, it is possible the influence of non-studied intervening variables, considering that this study is transversal. The number of pregnancies was included, considering that a patient with previous pregnancies could present a larger variation on her oral health, because the presence of large quantities of steroidal hormones causes a series of changes resulting in gingival tissues less resistant to periodontal pathogenic bacteria, causing a deterioration of oral conditions in women (6).

The present study included several limitations due to the fact that, considering the participation of first trimester pregnant women, many presented hyperemesis gravidarum, complicating data collection, and extending time for study completion. Because of this, it is recommended to work with a larger sample size and multicentric, in order to compare with results from this study, as well as include other factors that may influence the relation between periodontal index and saliva pH in pregnant women.

It is important to determine factors influencing the development of periodontal disease in order to take actions, both preventive and therapeutic. If these risk and protection factors are better known for periodontal disease, we could implement strategies to improve general health condition and quality of life.

## Conclusions

According to results from this study, it is concluded that first trimester pregnant women with lower saliva pH, exhibited a higher deterioration of periodontal condition. Among patients evaluated, code 3 of CPI was the most frequent (periodontal pockets smaller than 5.5 mm). No correlation was found between periodontal index and saliva pH, when patients were segregated by age group or number of pregnancies.
